# COVID-19-associated secondary sclerosing cholangitis with liver transplantation

**DOI:** 10.1007/s00428-024-03753-4

**Published:** 2024-03-25

**Authors:** Anne Kristin Fischer, Dirk Stippel, Ali Canbay, Dirk Nierhoff, Michael Thomas, Jan Best, Reinhard Büttner, Uta Drebber

**Affiliations:** 1 Institute of Pathology, University of Cologne, Kerpener Str. 62, 50937 Cologne, Germany; 2 Department of Surgery, University of Cologne, Kerpener Str. 62, 50937 Cologne, Germany; 3 Department of Internal Medicine, Transplant Medicine, Gastroenterology and Hepatology, University of Bochum, In Der Schornau 23–25 44892, Bochum, Germany; 4 Department of Internal Medicine, Gastroenterology and Hepatology, University of Cologne, Kerpener Str. 62, 50937 Cologne, Germany

**Keywords:** Liver pathology, COVID-19 infection, Secondary sclerosing cholangitis, Vasculopathy, Liver transplantation

## Abstract

We report on two cases of orthotopic liver transplantation (OLTX) due to SARS-Cov2-associated secondary sclerosing cholangitis (SSC) following long-term artificial respiration and extra-corporal membrane oxygenation in intensive care. Under these conditions, SSC is a rapidly progredient biliary disease featuring degenerative cholangiopathy, loss of bile ducts, ductular and parenchymal cholestasis, biliary fibrosis, and finally cirrhosis. Reduced perfusion and oxygenation of the peribiliary plexus, severe concurrent infections, and secondary medico-toxic effects appear to play a crucial role in the pathogenesis of the disease. A direct cytopathic effect of SARS-Cov2 on endothelial cells followed by thrombosis and fibrosing obliteration in all parts of the vascular bed of the liver may enhance the virus-associated liver disease and particularly SSC.

## Introduction

The coronavirus pandemic began in 2019, affecting about 760 million people and resulting in nearly 6.9 million deaths worldwide [1]. After SARS-Cov2-induced pneumonia affecting the lungs, the liver is the second most affected organ, with elevated liver enzymes indicating liver cell necrosis reported in 69 to 76% of patients [2–5], especially in patients admitted to intensive care. This liver disease can be transient, but cases with a dismal prognosis requiring liver transplantation are increasingly observed. To date, about 30 cases of COVID-19-induced severe biliary tract disease have been described worldwide in the literature. Critical pathogenetic factors of COVID-19-associated cholangiopathy may include direct virus-induced cytopathy; insufficient oxygenation of the peribiliary plexus due to pulmonary disease; the conditions of invasive mechanical ventilation; concurrent infection, with septicaemia potentially resulting in disseminated intravascular coagulopathy (DIC) due to a general severe inflammatory reaction with a so-called ‘cytokine storm’; and secondary medico-toxic effects [5–7]. Furthermore, local pro-coagulant conditions due to virus-induced endothelial damage and secondary vascular changes have been the focus of recent publications [5–9]. Awareness and recognition of fatal COVID-19-associated SSC in time is crucial to increase the chance of providing proper treatment, including OLTX.

## Material and methods

### Ethics statement

Informed consent was obtained from all patients from whom tissue samples were included in the study.

### Histology

Liver specimens were fixed in neutral buffered formalin and embedded in paraffin. Histopathological analysis was performed on histochemical stainings (H&E, PAS, PAS-Diastase, Sirius red, Elastica van Gieson, Gomori, Prussian Blue, and Rhodanine) and on immunohistochemical stainings (Keratin 7, CD68, Glutamine synthetase, CD34, and CD31) according to standard procedures. All stainings were conducted automatically with the Leica Bond-MAX automated system (Leica Biosystems, Germany).

## Case reports and results

Both cases were selected from the routine surgical and consult files from the tertiary care centres of the university clinics of Cologne and Bochum.

ERCP in both cases found severe SSC with largely destroyed intrahepatic biliary ducts obliterated by biliary casts (Fig. 2A). *Serological diagnostics* are described in Table 1.

**Table 1:** Demographic, clinical, serological, and radiological characteristics

Demographic and clinical, radiological, and histopathological characteristics	Patient 1	Patient 2
Age	58	59
Gender	Male	Male
Ethnicity	Middle Eastern descendent	Caucasian descendent
Previous diseases/comorbidities	Hypertension	Extracapillary-proliferative glomerulonephritis, most likely Henoch-Schönlein purpura, with kidney transplantation in 2019, and medical immunosuppression
Previously known liver disease	None	Hepatorenal syndrome after kidney transplantation in 2019, upper gastrointestinal bleeding due to oesophageal varices, two-vessel coronary heart disease, heart insufficiency NYHA^+^ III (LVEF^++^ 35%), COPD^+++^/bronchial asthma overlap, tertiary hyperparathyroidism, insulin-dependent diabetes mellitus type II, arterial hypertension, deep vein thrombosis of the lower leg
Prone position	Yes	Yes
Tracheotomy	Yes	Yes
Extracorporeal membrane oxygenation	Yes	Yes
Complications of ventilation and intensive care	Oesophageal sphincter stenosis, secondary bacterial infection and septic shock (*Enterobacter cloacae*, *Klebsiella pneumoniae*), high parietal lacunar brain lesion	Acute deterioration of the kidney transplant (03/2023), hepatic encephalopathy West Haven grade I–II, severe accompanying bacterial and viral infections (nosocomial acquired pneumonia with *Mycoplasma pneumoniae* (03/2023), EBV viraemia (03/2023), urinary tract infection with *Pseudomonas aeruginosa*, *Enterobacter cloacae*, and *Candida albicans* (05/2023)
Medication	None	Immunosuppression: tacrolimus, mycophenolate-mofetil, prednisolone, and basiliximab Anti-infective therapy: Linezolid
ERCP findings	Secondary sclerosing cholangitis with rarefied and partially strongly calibre variable intra- and extrahepatic bile ducts. Biliary casts. No predominant stenosis. Normal wide common hepatic bile duct	Secondary sclerosing cholangitis with largely destructed intrahepatic biliary system
Liver specific serological findings†	ALT: 42.9↑‡ U/L (R** < 41), AST: 47.1↑ U/L (R** < 50), γGT: 128↑ U/L (R** < 10–71), AP: 158↑ U/L (R**40–129), total bilirubin: 0.5 mg/dl (R** < 1.0), CHE: 2.19↓⁕ kUL (R**5.3–12.9), CRP: < 0.425 mg/dl (R** < 0.5)	ALT: 60↑ U/L (R** < 50), AST: 77↑ U/L (R** < 50), γGT: 1864↑ U/L (R** < 60), AP: 1476↑ U/L (R**40–129), total bilirubin: 19.0↑ mg/dl (R** < 1.2), direct bilirubin: 16.4↑ mg/dl (R** < 0.3), indirect bilirubin: 2.6↑ mg/dl (R** < 1.0) CHE: 3.4↓ kUL (R**5.3–12.9), CRP: 50.4↑ mg/dl (R** < 5.0)
Autoimmune diagnostics	Negative autoimmune diagnostic (ANA, LKM-1, AMA, c-ANCA, p-ANCA)	Negative autoimmune diagnostic (ANA, ASMA, LKM-1, LC-1, SLA, AMA, p-ANCA)

^+^*NYHA* New York Heart Association, ^++^*LVEF* left ventricular ejection fraction, ^+++^*COPD* chronic obstructive pulmonary disease, †Before OLTX, ‡↑ = elevated, *R = reference, ⁕↓ = decreased

Case 1: A 58-year-old man underwent OLTX after developing SSC with liver failure (MELD-Score: 12, Child–Pugh Score: B) after SARS-Cov2 infection 3 months before (he had received two vaccination doses). During his stay in ICU, he was treated with invasive long-term ventilation and extracorporeal membrane oxygenation (ECMO) therapy. He developed oesophageal sphincter stenosis following tracheotomy and suffered from a severe secondary bacterial infection (with *Enterobacter cloacae* and *Klebsiella pneumoniae*) and septic shock. Furthermore, high parietal lacunar ischaemic brain lesions occurred. His only pre-existing medical condition was arterial hypertension.

**Fig. 1:**
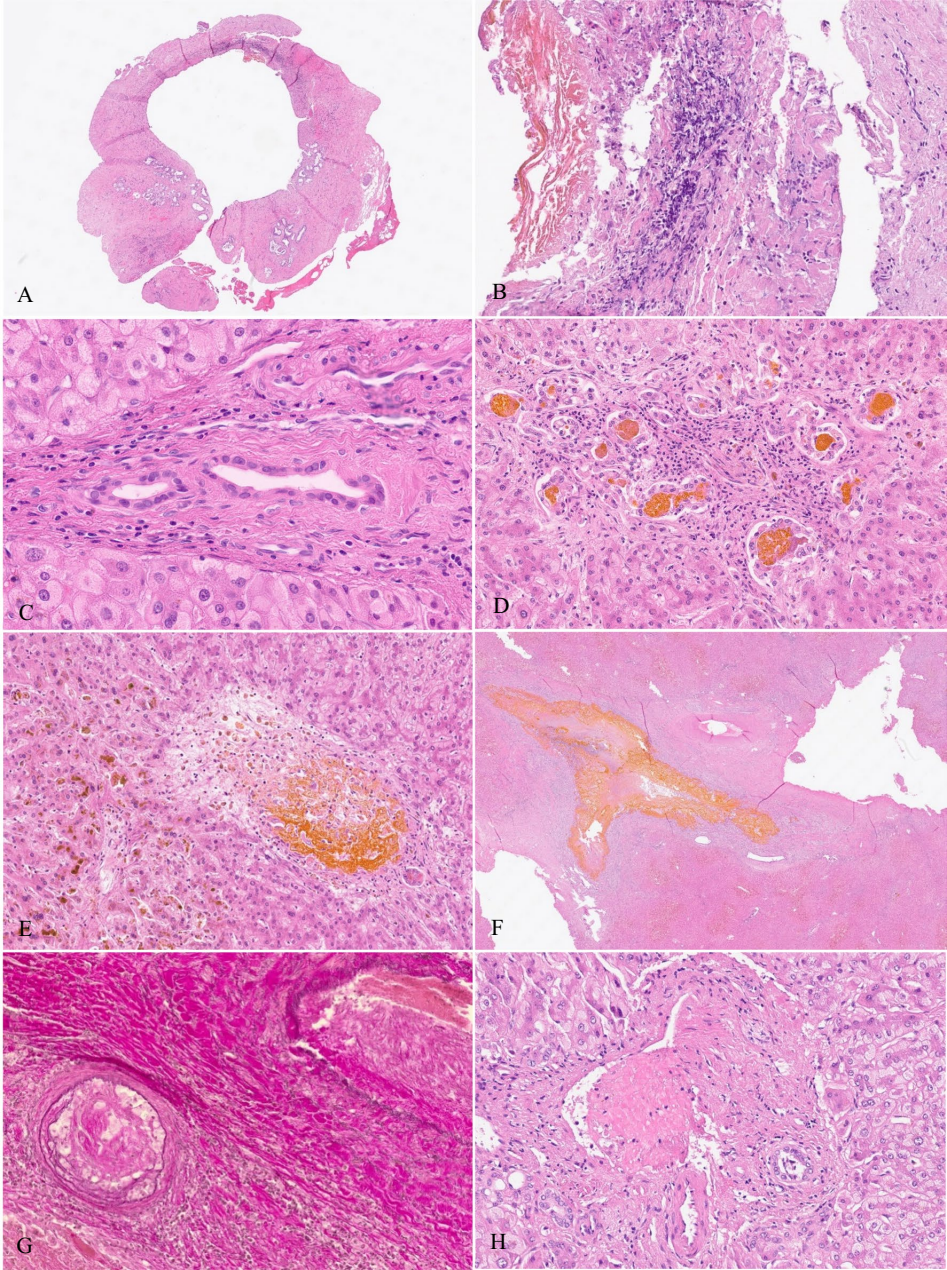
Patient 1: **A**, **B** The common hepatic duct shows a cushion-like transmural fibrosis. Instead of an epithelial lining (**A**) there are focal necroses with bile impregnation (**B**). **C** An interlobular bile duct with a degenerated epithelial lining embedded in fibrotic portal stroma. **D** Bile casts in periportal neoductules with degenerative changes of the nuclei. **E** Small bile infarct including a portal tract, strong centrilobular bilirubinostasis. **F** Large bile infarct with obliterating destruction of a septal bile duct. **G** An almost obliterated hepatic artery, plugged by foamy macrophages, fibroblasts, and fibrin (right) and a portal vein with an organised thrombus (left) (EvG). **H** Obliterating fibrin thrombus in a peripheral portal vein

Histologically, the common hepatic duct showed a cushion-like fibrosis (Fig. 1A). Focal necroses with epithelial loss and bile impregnation were also observed (Fig. 1B). The liver comprised porto-portal biliary fibrosis with marked fibro-ductular reaction surrounding portal tracts. Many bile ducts and neoductules were degenerated, showing defects, apoptoses, nuclear atypia, and flattening or swelling of the epithelial lining (Fig. 1C). Peripheral bile ducts and neoductules were occluded by bile casts (Fig. 1D), and others narrowed by a thickened fibrous wall. Small bile infarcts destroyed portal tracts and the surrounding parenchyma (Fig. 1E). Large bile infarcts led to obliterative destruction of septal bile ducts (Fig. 1F). Particularly in the periportal parenchyma, there was feathery hydropic degeneration of hepatocytes, some of them containing Mallory-Denk-bodies or copper granules. Severe bilirubinostasis was found in the centrilobular regions. Nests of xanthomatous macrophages were distributed randomly in the parenchyma. Vasculopathy was observed multifocally: Hepatic arteries and portal veins were partially or completely obliterated by fibrin clots or foamy macrophages (Fig. 1G and H).

Case 2: A 59-year-old man underwent OLTX due to SSC with liver cirrhosis after initially clinically manifesting SARS-Cov2 infection 4 months prior (initial ct-value, 23), followed by acute respiratory distress syndrome (ARDS). He had received four vaccination doses before and was infectious for 10 days. His prolonged stay in ICU was complicated by hepatic encephalopathy, bacterial and viral infections (nosocomial *Mycoplasma pneumoniae* pneumonia and EBV viraemia), and urinary tract infection (*Pseudomonas aeruginosa*, *Enterobacter cloacae*, and *Candida albicans*), as well as failure of his renal transplant. His past medical history included hepatorenal syndrome following kidney transplantation due to extracapillary-proliferative glomerulonephritis 4 years before COVID-19 infection; upper gastrointestinal bleeding due to oesophageal varices; two-vessel coronary heart disease; heart insufficiency NYHA III; COPD/bronchial asthma overlap; tertiary hyperparathyroidism; insulin-dependent diabetes mellitus type II; and arterial hypertension.

**Fig. 2:**
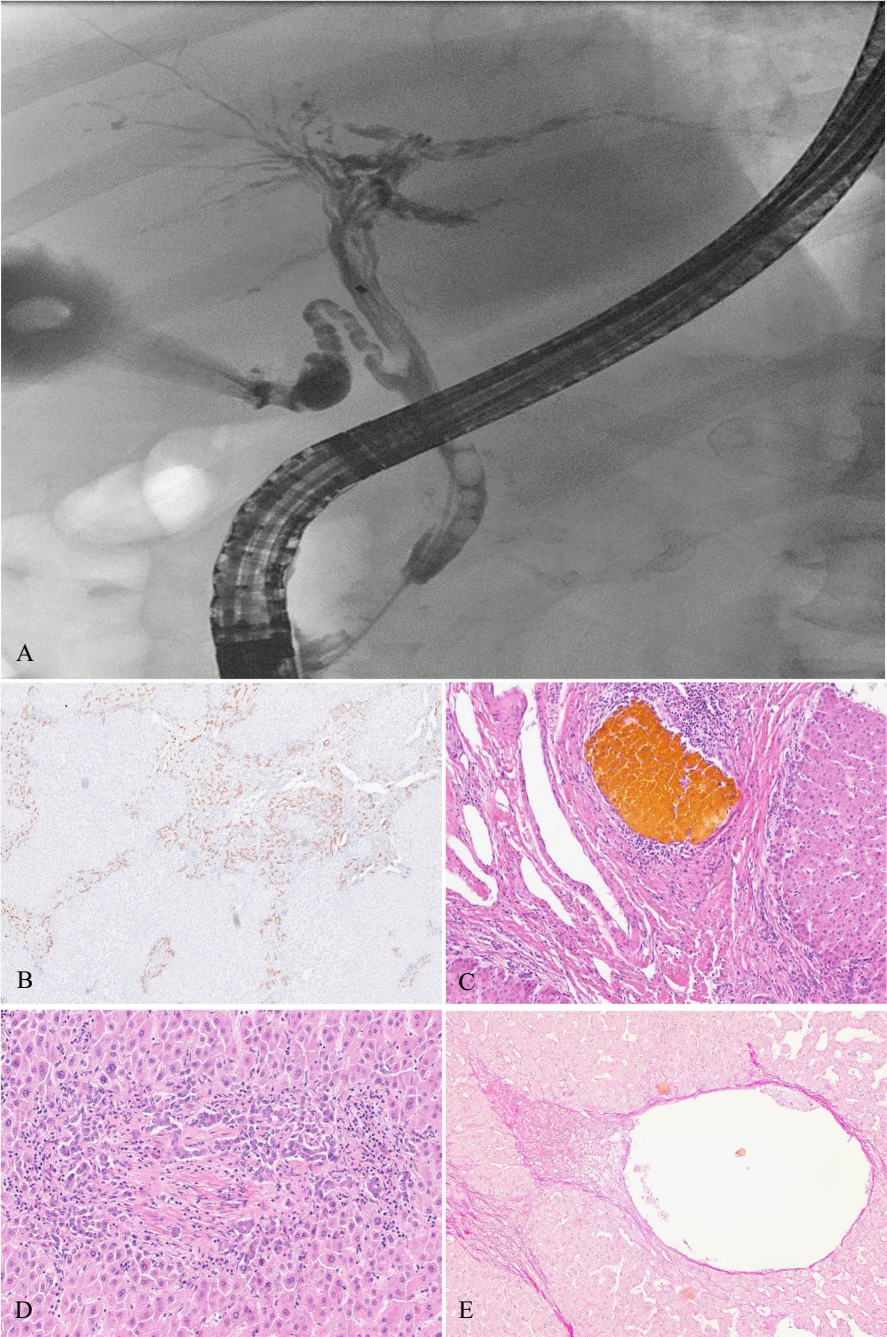
Patient 2: **A** ERCP imaging representing far progredient secondary sclerosing cholangitis with a largely destructed intrahepatic biliary duct system. **B** Biliary cirrhosis with fibroductular reaction along the porto-portal septa (CK7). **C** A small septal bile duct obliterated by a bile clot surrounded by a dense lymphocytic infiltration. The epithelial lining is lost. **D** Fibrosclerotic peripheral portal tract surrounded by a fibroductular reaction; preformed bile duct, arterioles, and portal vein are lost. **E** Partially fibrotic obliterated central vein with foamy macrophages

Histologically, the liver transplant displayed heterogeneous liver cirrhosis: in areas with a biliary cirrhosis pattern, the portal tracts were closely connected by fibro-ductular septa surrounding the parenchyma (Fig. 2B) or more or less extinct fibrotic transformed parenchymal residues. Other areas had developed regenerative nodules. Some septal bile ducts were obliterated by bile clots and lacked an epithelial lining (Fig. 2C). Many portal tracts were obliterated by dense fibrosclerosis without arteriolar or porto-venous structures and preformed bile ducts; they were surrounded by a fibro-ductular reaction (Fig. 2D). Occasionally, feathery degenerated periportal hepatocytes contained copper granules. Some central veins and neighbouring sinusoids were occluded by a loose post-thrombotic fibrosis with interspersed macrophages (Fig. 2E). The common hepatic bile duct showed a dense lympho-plasmocytic and granulocytic infiltration of the mucosal stroma and fibrosis of the wall.

Follow up: Following successful OLTX and an uncomplicated postoperative course, both patients recovered well, and the transplant liver function was satisfactory with continuously regredient liver serum parameters.

## Discussion

The impact of SARS-Cov2-induced acute respiratory syndrome on the liver is reinforced by an interplay between direct viral cytotoxicity on hepatocytes and cholangiocytes, ICU-associated reduced systemic and arterio-hepatic blood flow, medico-toxic side effects, and a severe general inflammatory reaction. The dysregulation of the splanchnic blood flow and oxygen supply is aggravated by an alteration of the hepatic vascular bed. Steatosis and unspecific mild necro-inflammatory infiltration are commonly reported findings [10, 11]. COVID-19 infection can primarily affect the liver parenchyma. Higher elevations in AST and ALT compared to γGT and AP were observed in COVID-19-associated hepatopathy, with only 12% of patients suffering from cholestasis [3, 4]. However, severe hepatocellular necrosis appears to be a secondary effect of disrupted hepatic blood supply due to thrombosis, infarct, or DIC, which may be directly enhanced by virus-induced vasculopathy, or indirectly in cases with a severe disease course.

Nevertheless, the biliary tract can also be damaged. COVID-19-associated SSC is increasingly described in the literature [5–7, 12–14]. Cholangiocytes and liver sinusoidal endothelial cells (LSECs) express the angiotensin-converting enzyme 2 (ACE2) receptor at a high density on their surface, which is a viral target [9, 15]. The biliary system can therefore become the focus of hepatopathy, and biliary diseases with clinico-serological similarities to primary sclerosing cholangitis (PSC) can occur.

In contrast to the majority of previous case reports, which describe only mild bile duct injury, both our patients have SSC on ERCP imaging, mirrored by extremely high levels of γGT and AP. Neither autoantibodies signalled an autoimmune liver disease nor could parameters for a virus-induced hepatitis be detected on broad serological follow-up analyses.

Our first patient demonstrated a pattern of biliary fibrosis associated with ischemic cholangiopathy of the large and peripheral bile ducts, as well as severe parenchymal bilirubinostasis and cholestasis. Aggregates of neutrophilic granulocytes in a large bile infarct could indicate a former source of biliary septicaemia. This case resembles the so-called ‘secondary sclerosing cholangitis in critically ill patients’ (SSC-CIP) in a progredient state following complex long-term treatment in the ICU.

The liver pathology in the second patient may have been affected by their complex medical history prior to COVID-19 infection. The liver explant showed a striking picture of a biliary cirrhosis with porto-portal septal fibrosis and a massive neoductular reaction, as well as the characteristic features of SSC on ERCP imaging. Bile clots and portal fibrosclerosis with loss of interlobular ducts were findings in this patient which characterise sclerosing cholangitis in critically ill patients (SSC-CIP).

Remarkably, vascular changes of small arteries, portal, and centrilobular veins, as well as adjacent sinusoids with obliterating thrombi in different stages of organisation, were observed in both patients, as reported by other authors [5–7, 12–14]. Direct endothelial damage and locally altered coagulation during the course of infection have to be considered cofactors for COVID-19-induced liver damage in general and particularly in the development of COVID-19-associated SSC. This consideration should be made in addition to the general impairment of the blood circulation which occurs during complicated ICU treatment. In the presented cases of late-stage liver disease following COVID-19 infection months prior, no morphological or serological signs of an underlying necro-inflammatory process were detected.

In conclusion, COVID-19-associated SSC is a rare, severe, and often fatal disease which is different to primary inflammatory bile duct disorders such as PSC and primary biliary cholangitis (PBC), but with analogies to SSC-CIP. Both presented cases were characterised by prolonged respiratory insufficiency treated by ECMO; both were complicated by septic shock or bacterial pneumonia, which contribute to insufficient oxygenation of the bile duct supporting blood flow; and the course of one case was aggravated by a pre-existing severe medical burden before COVID-19 infection. Additionally, a COVID-19-specific alteration of endothelial cells mediated by the ACE-receptor as an anchor protein for viral entrance, as well as local pro-coagulative conditions followed by vasculopathy and thrombosis, may enhance the liver damage in general and specifically the vulnerability of the bile duct epithelium.

## Data Availability

The data used and analysed during the current study are available from the corresponding author on reasonable request.

## Abbreviations

*ALT*: Alanine aminotransferase
*AMA*: Antimitochondrial antibody
*ANA*: Antinuclear antibody
*AP*: Alkaline phosphatase
*ARDS*: Acute respiratory distress syndrome
*AST*: Aspartate aminotransferase
*cANCA*: Antineutrophilic cytoplasmatic antibody
*CHE*: Cholinesterase
*CK7*: Cytokeratin 7
*COPD*: Chronic obstructive pulmonary disease
*COVID-19*: Coronavirus disease 2019
*Ct*: Cycle threshold
*DIC*: Disseminated intravascular coagulopathy
*EBV*: Epstein Barr virus
*ECMO*: Extracorporeal membrane oxygenation
*ERCP*: Endoscopic retrograde cholangiopancreatography
*EvG*: Elastica van Gieson
γGT: Gamma glutamyl transferase
*ICU*: Intensive care unit
*LC-1*: Anti-liver cytosol antibody
*LKM-1*: Anti-liver kidney antibody
*LSECs*: Liver sinusoidal endothelial cells
*LVEF*: Left ventricular ejection fraction
*MELD-Score*: Model for End-Stage Liver Disease Score
*NYHA*: New York Heart Association
*OLTX*: Orthotopic liver transplantation
*pANCA*: Perinuclear antineutrophilic cytoplasmatic antibody
*PAS*: Periodic acid Schiff reaction
*PBC*: Primary biliary cholangitis
*PSC*: Primary sclerosing cholangitis
*SARS-Cov2*: Severe acute respiratory syndrome
*SLA*: Soluble liver antigene
*SSC-CIP*: Sclerosing cholangitis in critically ill patients
